# A FRET-based screening method to detect potential inhibitors of the binding of CNNM3 to PRL2

**DOI:** 10.1038/s41598-020-69818-x

**Published:** 2020-07-30

**Authors:** Faji Cai, Yichen Huang, Mengqi Wang, Minxuan Sun, Yimeng Zhao, Motoyuki Hattori

**Affiliations:** State Key Laboratory of Genetic Engineering, Collaborative Innovation Center of Genetics and Development, Department of Physiology and Biophysics, School of Life Sciences, Fudan University, 2005 Songhu Road, Yangpu District, Shanghai, 200438 China

**Keywords:** High-throughput screening, Screening, Biological fluorescence, Fluorescence imaging

## Abstract

The cyclin M (CNNM) family of Mg^2+^ transporters is reported to promote tumour progression by binding to phosphatase of regenerating liver (PRL) proteins. Here, we established an assay for detection of the binding between the cystathionine-beta-synthase (CBS) domain of human CNNM3 (a region responsible for PRL binding) and human PRL2 using fluorescence resonance energy transfer (FRET) techniques. By fusing YPet to the C-terminus of the CNNM3 CBS domain and CyPet to the N-terminus of PRL2, we performed a FRET-based binding assay with purified proteins in multiwell plates and successfully detected the changes in fluorescence intensity derived from FRET with a reasonable *K*_d_. We then confirmed that the addition of non-YPet-tagged CNNM3 and non-CyPet-tagged PRL proteins inhibited the changes in FRET intensity, whereas non-YPet-tagged CNNM3 with a mutation at the PRL2-binding site did not exhibit such inhibition. Furthermore, newly synthesized peptides derived from the CNNM loop region, with the PRL-binding sequences of the CNNM3 CBS domain, inhibited the interactions between CNNM3 and PRL2. Overall, these results showed that this method can be used for screening to identify inhibitors of CNNM-PRL interactions, potentially for novel anticancer therapy.

## Introduction

Cyclin M (CNNM) is a Mg^2+^ transport protein^1–3^ that was originally named after a domain similar to one in cyclin^4^ but has no reported cell cycle-related function. Several structures of its soluble region have been determined by X-ray crystallography^5–11^. The human body contains four subtypes of CNNM family proteins, CNNM1-CNNM4, and mutations of CNNM can cause abnormalities in intracellular Mg^2+^ homeostasis that lead to severe symptoms. For example, CNNM2 is reported to be the gene responsible for familial primary hypomagnesemia^12^, and Jalili syndrome is caused by CNNM4 mutation^13,14^. CNNM2-mutated mice exhibited viviparous lethality and significant Mg^2+^ reabsorption deficiency^15^.

Recently, CNNM functions related to cancer have attracted attention. Phosphatase of regenerating liver (PRL) is a small 20 kDa protein^16^ with only a tyrosine phosphatase domain. In 2001, a high expression level of PRL3 in intestinal tumour metastases was reported^17^. The regulatory role of the binding of PRL to CNNM in Mg^2+^ transport was subsequently reported^18,19^. There are three human PRLs (PRL1-PRL3), all of which bind to CNNM proteins in any combination^18,19^. Crystal structures of the CNNM CBS domain in complex with PRL proteins have been reported, revealing the structural basis for the CNNM-PRL interactions^5,11,19,20^. The CNNM3 cystathionine-beta-synthase (CBS) mutation D426A is one of the most severe mutations, preventing CNNM3/PRL2 complex formation^21^. In addition, phosphorylated PRL does not bind to CNNM^19^.

What is the result of the inhibition of Mg^2+^ export caused by the binding of PRL to CNNM? Both CNNM2-deficient mice and CNNM4-deficient mice showed defective Mg^2+^ (re)absorption^15,19^, but no oncogenesis was observed. However, additional CNNM4 knockout promotes tumour progression (invasion) of intestinal polyps in an APC-hetero-deficient background^19^. PRL overexpression increases the intracellular Mg^2+^ concentration, accompanied by an increase in the amount of ATP^15^. These cells show a normal proliferation rate even with low glucose content (2 mM), when most cells stop division. This suggests that the Mg^2+^ concentration affects energy metabolism. Furthermore, inhibition of the binding of PRL2 to CNNM3 decreased tumour progression^22^.

Hence, the CNNM and PRL complex is a promising target for drug design to combat tumour progression and metastasis. To date, only a few chemical compounds targeting the interactions between CNNM and PRL proteins have been made available. For instance, thienopyridone and its derivatives have been characterized as PRL inhibitors^23,24^. Thienopyridone was shown to interrupt the binding between CNNM3 and PRL2 as well as reduce cancer cell proliferation^21^. However, it was also suggested that thienopyridone and its derivatives are potentially problematic due to their possible off-target effects^25^.

Here, we established a CNNM-PRL complex binding detection assay using purified proteins and fluorescence resonance energy transfer (FRET) techniques. Using this assay, we can quickly and accurately screen drug candidates.

## Results

### Construct design for detecting CNNM3 and PRL2 complex formation by FRET

**Figure 1:**
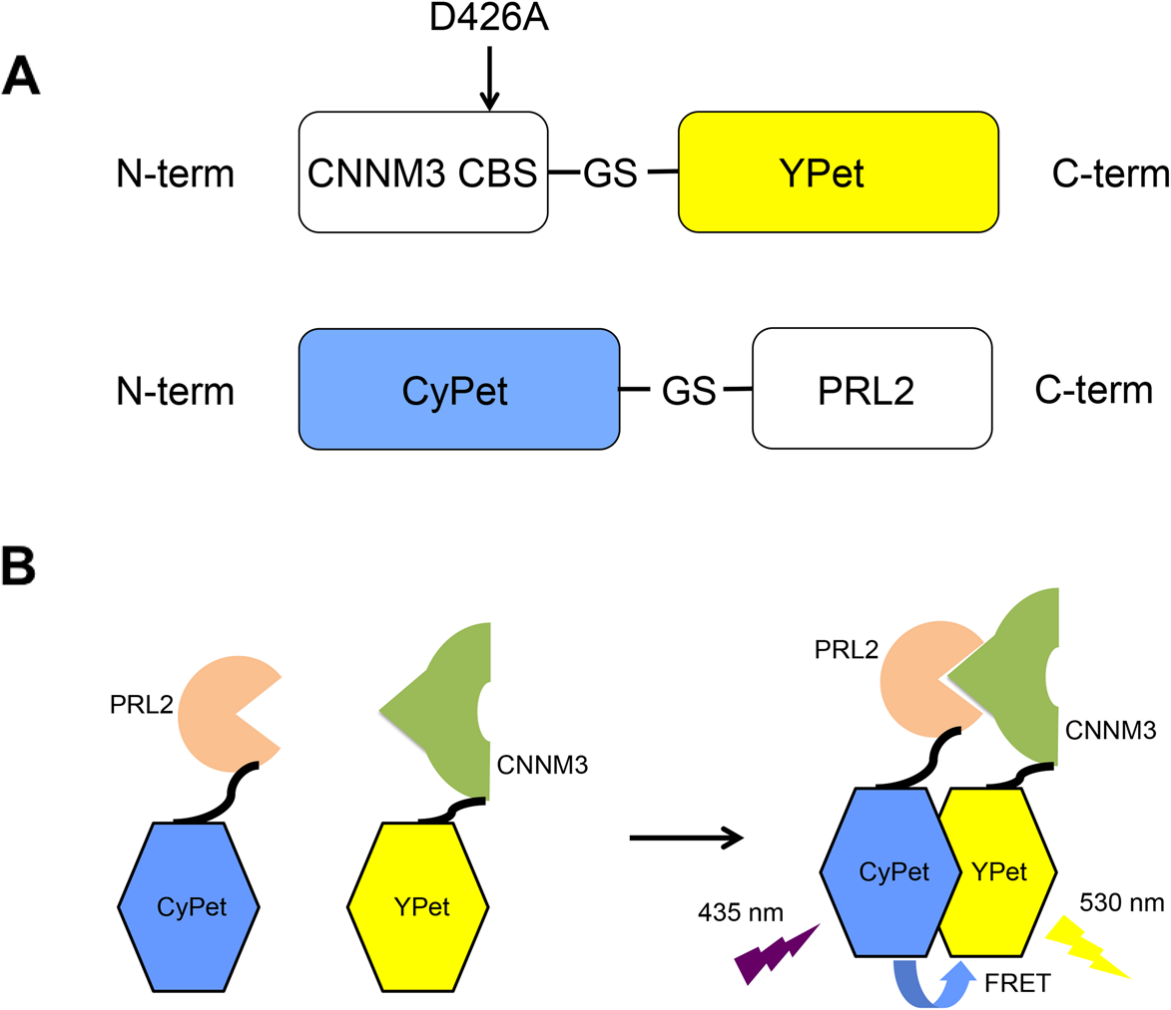
FRET binding model of the CNNM3 CBS domain and PRL2. (**A**) Cartoon depiction of construct design. (**B**) FRET model of the binding of CNNM3-YPet and CyPet-PRL2.

To design the CNNM3 and PRL2 constructs for FRET, we carefully examined the previously determined structure of the CNNM3 CBS domain and PRL2 (PDB ID: 5K22) and found that the distance between the C-terminus of the CNNM3 CBS domain and the N-terminus of PRL2 is approximately 35 Å, which is sufficient for observing FRET (10–100 Å)^26^. Thus, we added a YPet tag to the C-terminus of the CNNM3 CBS domain (residues 301–452) and attached a CyPet tag to the N-terminus of PRL2 (residues 1–167) (Fig. 1A). When CNNM3 and PRL2 form a complex, FRET should occur, and the related fluorescence should be detected; otherwise, no related fluorescence should be observed (Fig. 1B). After making these constructs, we purified each protein separately from *E. coli* culture as a recombinant protein (Supplementary Fig. 1).

### FRET-based assay to detect the binding of CNNM3 to PRL2

First, we performed spectral measurement for purified CyPet-PRL2/CNNM3-YPet (FRET emission), CyPet-PRL2 and CNNM3-YPet excited at 435 nm (Supplementary Fig. 2) and observed a drastic increase in FRET efficiency due to the binding between the CNNM3 and PRL2 proteins.

**Figure 2:**
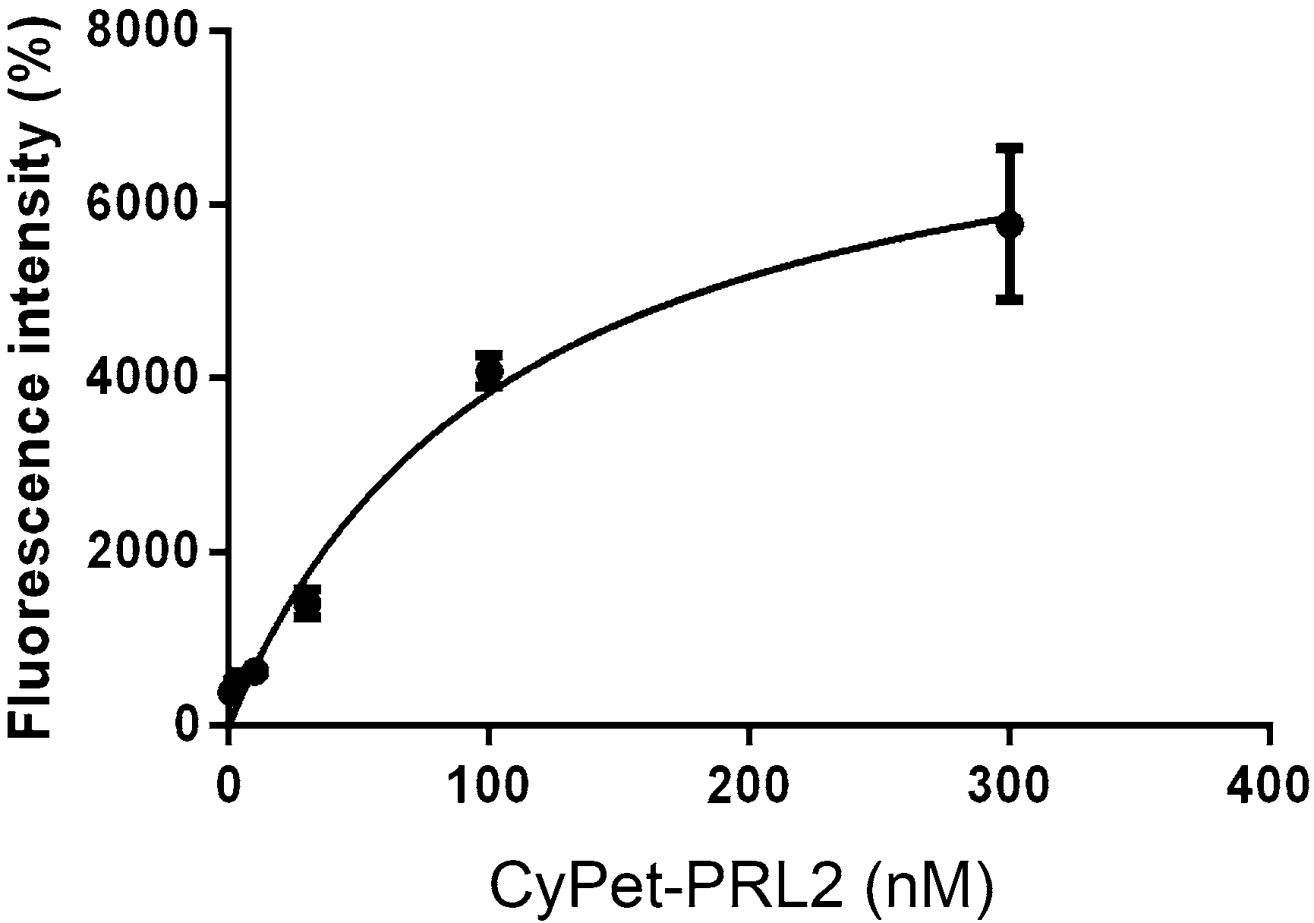
CNNM3-YPet binding to CyPet-PRL2 detected by FRET. FRET data regarding the binding of CNNM3-YPet to CyPet-PRL2. R^2^ = 0.9588, *n* = 6.

We then tested the binding of purified CNNM3-YPet and CyPet-PRL2 for FRET experiments. The FRET data showed that CNNM3-YPet successfully bound to CyPet-PRL2 with a *K*_d_ of 108 ± 16 nM (Fig. 2), which is comparable to the previously reported *K*_d_ value obtained by isothermal titration calorimetry (ITC) using non-fluorescence-tagged proteins^5^.

### Non-fluorescence-tagged CNNM3 abolished FRET-based binding

**Figure 3:**
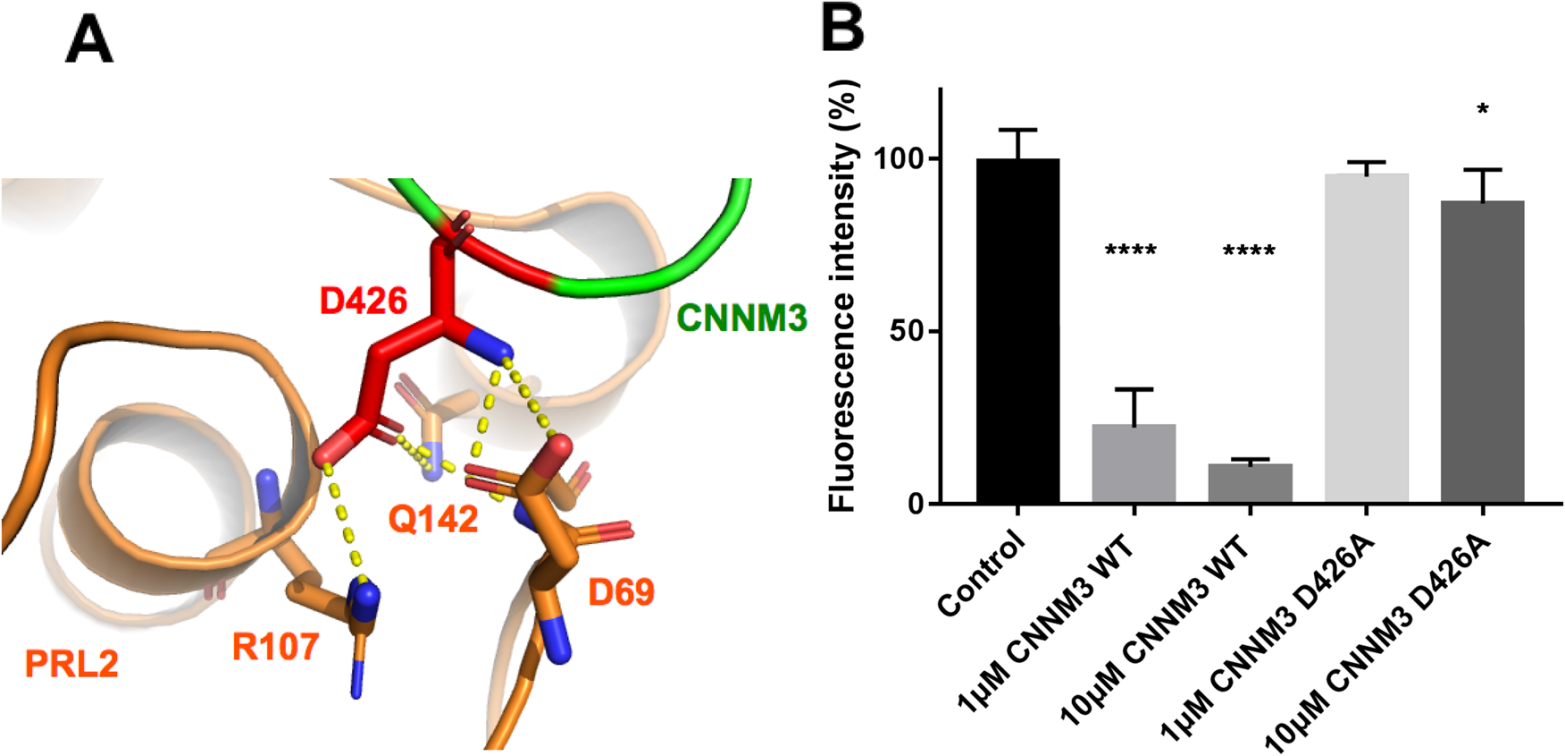
Inhibition of the binding of CNNM3-YPet and CyPet-PRL2 using non-YPet-tagged wild-type CNNM3 CBS and mutated CNNM3 CBS. (**A**) Close-up view of the CNNM3-PRL binding site (PDB: 5K22). (**B**) Non-YPet-tagged CNNM3 CBS-based inhibition test of the binding of CNNM3-YPet (200 nM) and CyPet-PRL2 (200 nM) using FRET. All data are expressed as the mean ± SE. *p < 0.05, ****p < 0.0001, by Tukey’s multiple comparison test, *n* = 6.

Since we aimed to establish a screening method for the binding and separation of CNNM3 and PRL2, our FRET-based assay needed to detect not only the binding but also the dissociation of CNNM3 and PRL2. Therefore, we tested whether the addition of inhibitory factors could abolish the FRET intensity in the assay. Non-fluorescence-tagged CNNM3 would be a promising candidate, as it would compete with YPet-tagged CNNM3 for binding to CyPet-tagged PRL2. Furthermore, the CNNM3 CBS domain D426A mutant with mutations at the PRL2-binding site (Fig. 3A) reportedly exhibits very weak binding to PRL proteins and would therefore be an ideal control sample for establishing the FRET-based assay system. The addition of non-YPet-tagged CNNM3 at concentrations of 1 and 10 μM abolished 77.7 and 89.2% of the FRET intensity, respectively, in our assay using CNNM3-YPet and CyPet-PRL2 (Fig. 3B). The addition of the CNNM3 CBS domain D426A mutant had little effect on the FRET intensity. Even at 10 μM, the CNNM3 CBS domain D426A mutant decreased only 12.9% of the FRET intensity (Fig. 3B), which is consistent with the previously reported *K*_d_ value obtained by ITC for the CBS domain D426A mutant^9^. These results showed that our FRET assay can also detect the binding and dissociation of CNNM3 and PRL2.

### Non-fluorescence protein-tagged PRLs abolished FRET intensity

**Figure 4:**
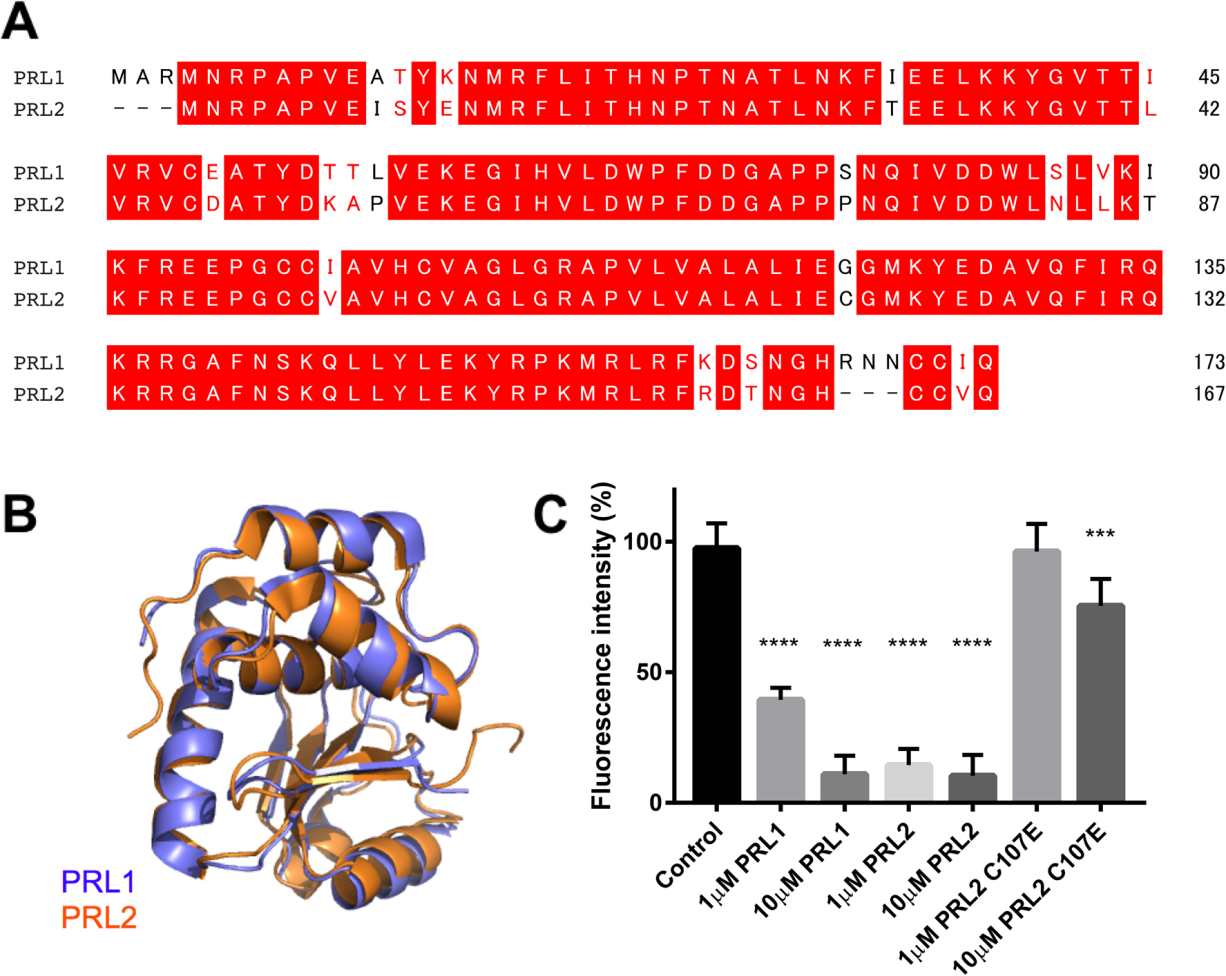
Inhibition of the binding of CNNM3-YPet and CyPet-PRL2 using non-CyPet-tagged PRL1 and PRL2. (**A**) Amino acid sequence alignment of human PRL1 (BAG70128.1) and human PRL2 (NP_001356788.1). Red boxes, identical residues; red letters, similar residues. (**B**) Structural comparison of PRL1 (blue, PDB: 1XM2) and PRL2 (orange, PDB: 5K22). (**C**) Non-CyPet PRL-based inhibition test of the binding of CNNM3-YPet (200 nM) and CyPet-PRL2 (200 nM) using FRET. All data are expressed as the mean ± SE. ***p < 0.001; ****p < 0.0001, by Tukey’s multiple comparison test, *n* = 6.

We then tested whether the addition of another component of the CNNM3-PRL2 complex, PRL, can also inhibit the binding between CNNM3 and PRL2. Purified non-fluorescence protein-tagged PRL1 and PRL2 were prepared and then added to the assay at concentrations of 1 and 10 μM. PRL1 and PRL2 share most of their sequences, with 88% identity and 93% similarity (Fig. 4A). Therefore, the structures of these two proteins are highly similar (Fig. 4B). In our FRET assay, the addition of these proteins had different inhibitory effects on complex formation. PRL1 inhibited complex formation by 61.6% and 90.0% at concentrations of 1 µM and 10 µM, respectively, while PRL2 inhibited complex formation by 85.5% and 89.7% at concentrations of 1 µM and 10 µM, respectively (Fig. 4C). We also tested the C107E mutant of PRL2; the equivalent mutation in PRL3 leads to greatly reduced binding affinity with CNNM3 (*K*_d_ of 2,800 nM)^5^. We observed that addition of the C107E mutant had little effect on the FRET intensity (Fig. 4C). Furthermore, we measured the spectrum of CyPet-PRL2 C107E/CNNM3-YPet (Supplementary Fig. 3). Compared to the spectrum of CyPet-PRL2 WT/CNNM3-YPet (Supplementary Fig. 2), we did not observe a significant change in the fluorescence intensity, which further validated our FRET assay.

### Effect of divalent cations and nucleotides on CNNM3-PRL2 binding

**Figure 5:**
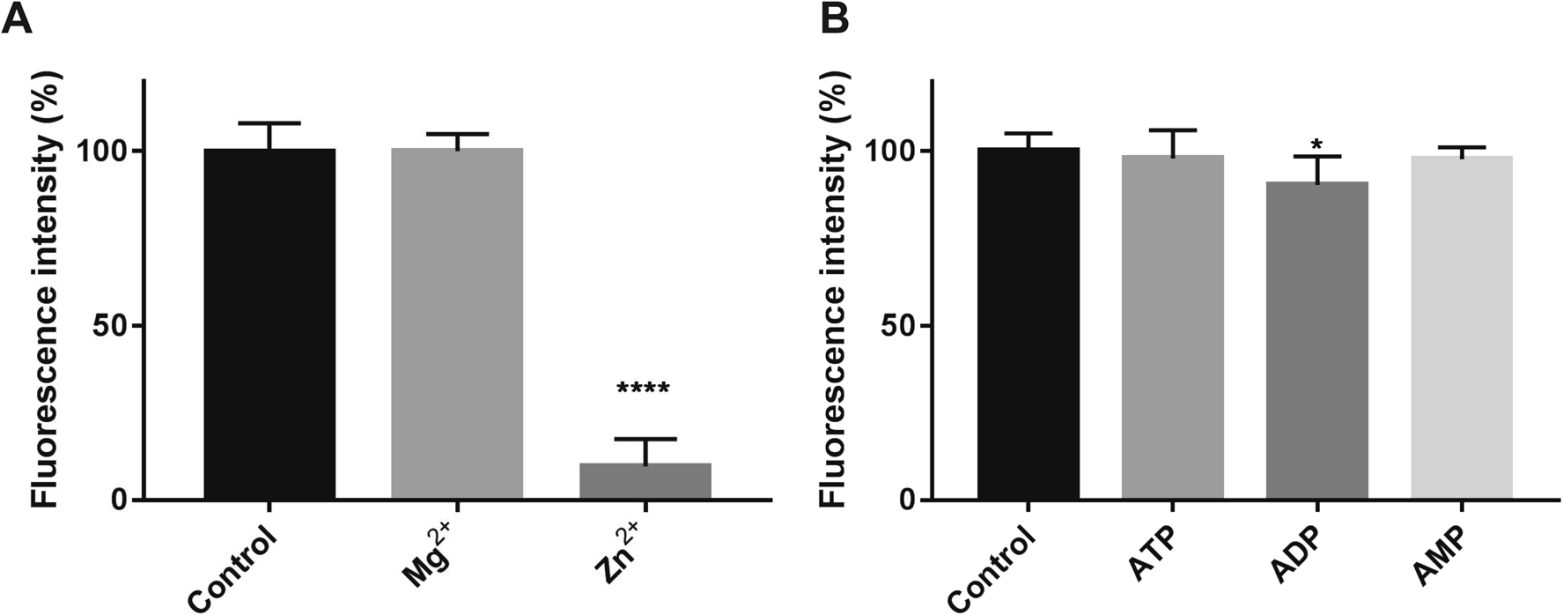
Effect of divalent cations and nucleotides on the FRET intensities. (**A**) Effect of divalent cations on the binding of CNNM3-YPet (200 nM) and CyPet-PRL2 (200 nM) using FRET. Control: No additional divalent cations; Mg^2+^: 10 mM Mg^2+^ was added; Zn^2+^: 10 mM Zn^2+^ was added. All data are expressed as the mean ± SE. ****p < 0.0001, by Dunnett’s multiple comparison test. *n* = 6. (**B**) Effect of nucleotides on the binding of CNNM3-YPet (200 nM) and CyPet-PRL2 (200 nM) using FRET. Control: No additional peptide. ATP: 3 mM ATP was added; ADP: 3 mM ADP was added; AMP: 3 mM AMP was added. All data are expressed as the mean ± SE. *p < 0.05, by *t*-test, *n* = 6.

We further tested the effects of divalent cations and nucleotides on CNNM3-PRL2 binding (Fig. 5). In our FRET assay, the addition of Mg^2+^ had no effect on the FRET intensity (Fig. 5A), whereas the addition of Zn^2+^ greatly deceased the FRET intensity by 90.3% (Fig. 5A), presumably due to the toxicity of a high concentration of Zn^2+^ ions towards the proteins. Furthermore, the addition of nucleotides had little effect on the FRET intensity (Fig. 5B). Only ADP exhibited a very weak effect on the FRET intensity, altering the intensity by 9.6%. Our result indicates that the interaction between CNNM3 and PRL2 is not regulated by the Mg-ATP complex, whereas a recent study indicated that the Mg-ATP complex is sensed by CNNM^8^.

### Peptides of the CNNM3 CBS loop sequence abolished the FRET signal

**Figure 6:**
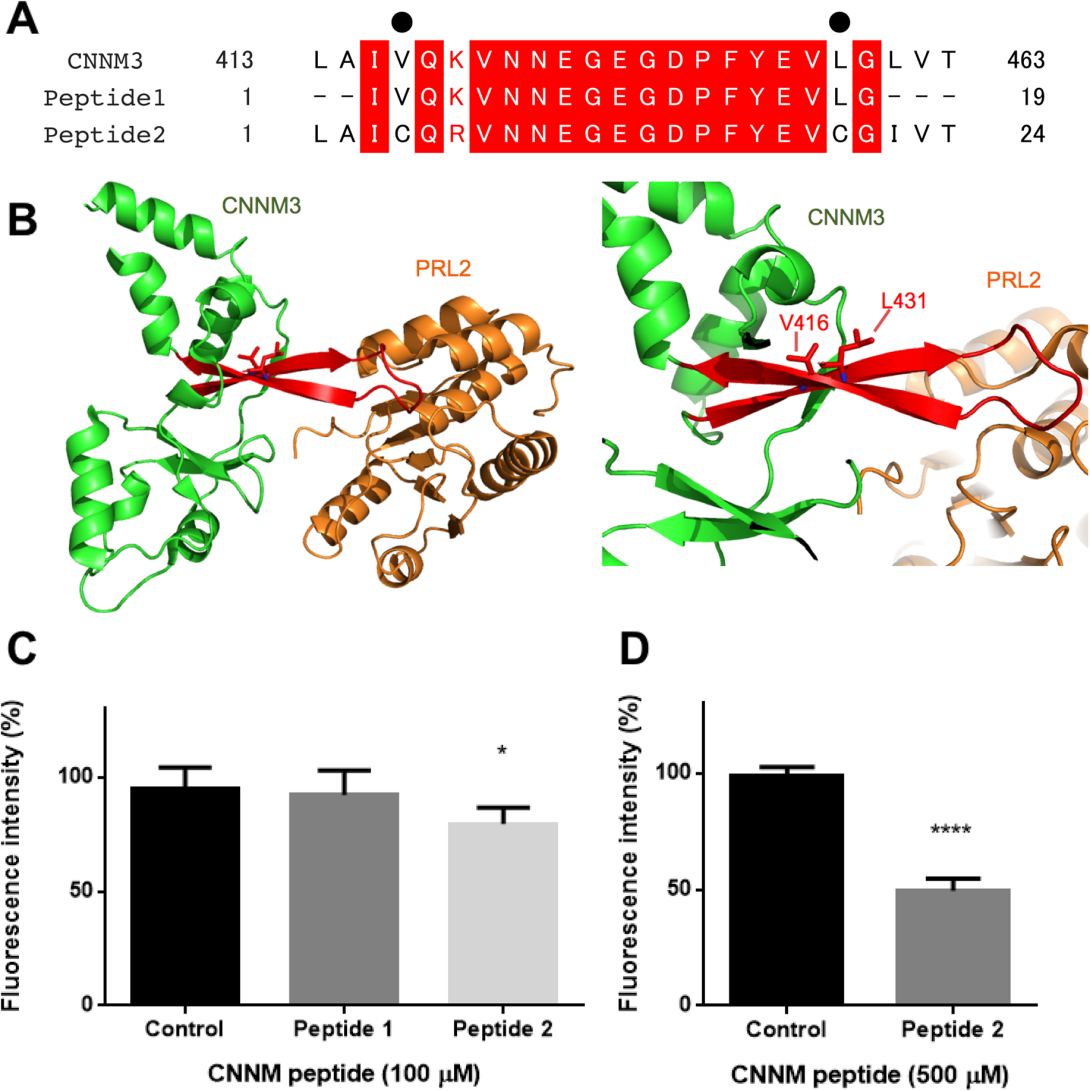
Inhibition of the binding of CNNM3-YPet and CyPet-PRL2 by CNNM peptides. (**A**) Amino acid sequences of synthesized peptides derived from the CNNM CBS loop. Black dots show the residues replaced by cysteine in peptide 2. Red boxes, identical residues; red letters, similar residues. (**B**) Location of CNNM peptides (red) in the CNNM3 structure (PDB: 5K22). V416 and L431 are replaced by cysteine residues in peptide 2. (**C**) CNNM peptide-based inhibition test of the binding of CNNM3-YPet (200 nM) and CyPet-PRL2 (200 nM) using FRET. Control: no additional peptide; peptide 1: 100 μM peptide 1 was added; peptide 2: 100 μM peptide 2 was added. All data are expressed as the mean ± SE. *p < 0.05, by Dunnett’s multiple comparison test. *n* = 6. (**D**) CNNM peptide 2-based inhibition test of the binding of CNNM3-YPet (200 nM) and CyPet-PRL2 (200 nM) using FRET. Control: no additional peptide. Peptide 2: 500 μM peptide 2 was added. All data are expressed as the mean ± SE. ****p < 0.0001, by *t*-test, *n* = 6.

To test whether inhibition by small molecules is detectable by our assay, we chose synthesized peptides mimicking approximately twenty amino acids (2.6 kDa) from the loop region of the CNNM3 CBS domain. This loop region is important for PRL binding, and its amino acid sequence is identical between CNNM3 and CNNM4. This region is also highly conserved in CNNM1 and CNNM2, showing 92% identity with CNNM3. Therefore, we synthesized two types of peptides (Fig. 6A) and measured their ability to inhibit complex formation (Fig. 6B). The shorter peptide (peptide 1, 100 μM) failed to interrupt complex formation (Fig. 6C). However, the longer peptide (peptide 2, 100 μM), with two residues mutated to cysteine residues to form an S–S bond for β-sheet structure retention (Fig. 6B), inhibited complex formation by 20% (Fig. 6C). Then, the peptide 2 concentration was raised to 500 μM, and the inhibition percentage increased to 50% (Fig. 6D).

**Figure 7:**
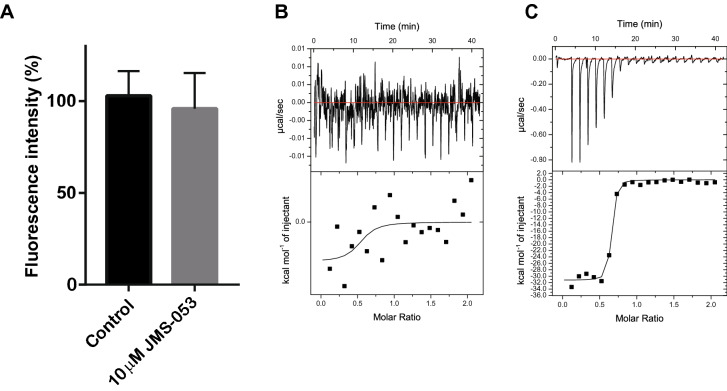
Evaluation of the JMS-053 effect on the interaction between CNNM3 and PRL2. (**A**) Effect of JMS-053 on the binding of CNNM3-YPet (200 nM) and CyPet-PRL2 (200 nM) using FRET. All data are expressed as the mean ± SE. *n* = 6. (**B**, **C**) ITC data on the PRL2 with JMS-053 (**B**) and CNNM3 CBS protein (**C**). Measurements were repeated twice, and similar results were obtained.

In addition to the peptides from the loop region of the CNNM3 CBS domain, we also tested a known PRL inhibitor, the thienopyridone derivative compound 13 (JMS-053), but we detected no effect of JMS-053 on the interaction between CNNM3 and PRL2 in our FRET-based assay (Fig. 7A). To verify this result, we also performed the ITC experiment with JMS-053, but there is no interaction between PRL2 and JMS-053 (Fig. 7B), whereas our ITC experiments properly detected the binding between the CNNM3 CBS domain and PRL2 (*K*_d_ = 15.2 ± 5.0 nM) (Fig. 7C). Our results indicate that the JMS-053 PRL inhibitor has no ability either to inhibit the interaction between CNNM3 and PRL2 or bind to PRL2.

## Discussion

In this study, we confirmed by a FRET-based assay that CNNM3 and PRL2 can form a complex, which is consistent with the results previously shown with non-tagged proteins^5,9,20^. Therefore, fluorescence protein tags did not influence the binding affinity, possibly because of the inserted linkers between the fluorescence tags and the target proteins (-GS- between CNNM3 CBS and CyPet, -GS- between YPet and PRL2) (Fig. 1A).

The most important aspect of our established FRET assay is that the FRET intensity changes reflect the actual binding between CNNM3-CyPet and YPet-PRL2. To show this, we conducted several tests for inhibition of complex formation. The addition of non-fluorescence protein-tagged PRL2/CNNM3 to fluorescence protein-tagged CNNM3/PRL2 pairs abolished the FRET signal (Figs. 3 and 4), suggesting that the observed FRET intensity correlates with the actual binding.

Figure 4 suggests that PRL2 may have a higher affinity for CNNM3 than PRL1. There was no report of the *K*_d_ value of the binding of CNNM3 with PRL1, and since PRL1 and PRL2 have some difference in their sequences (88% identity), the apparent differences in inhibitory effect between PRL1 and PRL2 may reflect these differences in amino acid sequence.

From the peptide inhibition test, we identified a primary model for peptide inhibitors of the CNNM3/PRL2 complex. Additionally, it was suggested that structure retention of the peptide is important for the inhibition of complex formation. However, the 500 μM peptide concentration that we tested is too high for practical use. Further mutations or modification of peptide 2 would be required to stabilize the structure of the CNNM peptide complex to increase the binding affinity and to provide membrane permeability. Peptide length optimization and the attachment of cell-permeable sequences such as TAT^27^ can be considered. With such improvements, our identified peptide model could be applied to a cell-based assay to investigate the magnesium exporter activity of CNNM or could be used in preclinical tests such as in vivo animal experiments examining tumour progression.

Compared to other methods, such as radioimmunoassays and ELISA, our FRET-based assay does not involve multiple steps of incubation and washing, which are typically time consuming. Therefore, as an advantage, our method can be performed with simple operations and can be easily automated.

On the other hand, as a disadvantage of our FRET-based assay, there are potentially some false positive hits obtained when proteins are denatured by the addition of small molecules and when small molecules themselves exhibit strong effects on the FRET reaction. This possibility can be excluded by combination with fluorescence size-exclusion chromatography (FSEC)^28^ to evaluate the protein condition. There is no need to prepare additional samples for the FSEC test since the samples measured with the FRET assay can be directly subjected to FSEC, which is also a simple method.

In conclusion, here, we have established a FRET-based assay to visualize the binding states of CNNM3 and PRL2. FRET-based binding detection had many applications and can be used for not only protein–protein interactions but also protein-drug interactions. By optimizing small-molecule hits in our FRET assay, we can obtain the ideal molecule in a straightforward way.

## Methods

### Plasmid construction

The CBS domain of human CNNM3 (XP_011509259.1, residue 301–452) was amplified from human cDNA by PCR and subcloned into a pET22c derivative with YPet^29^ at its C-terminus, and human PRL2 (NP_001356788.1, residue 1–167) was similarly cloned into a pET22c derivative with CyPet with monomeric mutation (A206K)^29,30^ at its N-terminus. Both CNNM3 and PRL2 have a GS linker between the fluorescent proteins and their fusion partners. For the expression of non-fluorescence protein-tagged proteins, the CBS domain of human CNNM3 and human PRL2 were subcloned into pET28a. The mutation D426A was introduced into the CNNM3 constructs by PCR and verified by DNA sequencing.

### Protein purification

The constructs were transformed into *Escherichia coli* Rosetta (DE3) cells and cultured in LB medium at 37 °C until the OD600 reached 0.5–0.8. Then, isopropylthio-beta-d-galactoside (IPTG) was added at a final concentration of 0.5 mM. Cultures were incubated at 18 °C for 20 h (for fluorescence protein-tagged proteins) or at 37 °C for 3 h (for non-fluorescence protein-tagged proteins). The cells were harvested by centrifugation at 5,000 rpm and resuspended in TBS (50 mM Tris–HCl (pH 8.0), 150 mM NaCl) supplemented with 1 mM PMSF and 1 mM 2-mercaptoethanol. The cells were then disrupted by liquid homogenization three times at 1,000 bar. Debris was removed by 1 h of centrifugation at 18,000 rpm, and the supernatant was mixed with Ni–NTA resin (Qiagen). The resin was washed with TBS supplemented with 30 mM imidazole and 1 mM 2-mercaptoethanol, and the proteins were eluted by TBS with 300 mM imidazole and 1 mM 2-mercaptoethanol. For PRL2 and YPet-PRL2, after overnight dialysis in TBS with 10 mM imidazole and 1 mM 2-mercaptoethanol, size-exclusion chromatography was performed by using a Superdex 200 10/300 GL column (GE Healthcare) in buffer containing 20 mM HEPES (pH 7.0), 150 mM NaCl, and 0.5 mM Tris (2-carboxyethyl) phosphine (TCEP). For CNNM3 CBS, CNNM3 CBS-CyPet and CNNM3 CBS (D426A), the overnight dialysis buffer was changed to 20 mM Tris–HCl (pH 7.0), 50 mM NaCl, and 1 mM 2-mercaptoethanol, and anion exchange chromatography was performed using a Hitrap 5 ml Q HP column (GE Healthcare).

### Fluorescence resonance energy transfer

FRET measurements were conducted in TBS (50 mM Tris–HCl (pH 8.0) and 150 mM NaCl). Proteins were mixed in 96-well plates and incubated at R.T. for 1 h. Fluorescence intensities were measured using an excitation wavelength of 435 nm and an emission wavelength of 530 nm with a Cytation 3 (BioTek).

The emission spectra were measured with each component at 200 nM using the Cytation 3 (BioTek) with 435 nm excitation (Supplementary Fig. 2), and the original data are shown in Supplementary Table 1. The FRET intensity was calculated by Eq. (1).

1 $$I_{{FRET}} = I_{A{\text{-}}raw} - D_{{leakage}} - A_{{direct}}$$

where *I*_*A-raw*_ is the intensity measured at 530 nm with 435 nm excitation, *D*_*leakage*_ is the leakage of the donor emission into the acceptor wavelength (530 nm) upon donor excitation, and *A*_*direct*_ is the direct excitation of the acceptor with the donor wavelength (435 nm).

To test the binding of CyPet-PRL2 and CNNM3 CBS-YPet (Fig. 2), 30 nM CyPet-PRL2 and serial dilutions of CNNM3 CBS-YPet (0, 1, 3, 10, 30, 100, 300 nM) were mixed.

The inhibition of complex formation was assayed by pre-incubating the binding partner (200 nM) with inhibitory factors (1, 10 μM) for 20 min at R.T., followed by addition of the other protein (200 nM) (Figs. 3B, 4C). The effects of divalent cations (10 mM) and nucleotides (3 mM) were also assayed similarly (Fig. 5).

The peptides for the peptide inhibition test were synthesized by Shanghai Dechi Biosciences (Fig. 6C,D). The peptide sequences were as follows: peptide 1, IVQKVNNEGEGDPFYEVLG; peptide 2, LAICQRVNNEGEGDPFYEVCGIVT (an S–S bond was formed between the cysteine residues, as verified by HPLC and mass spectrometry). The peptides were dissolved in TBS buffer at 10 mM before use.

FRET data were evaluated by subtracting backgrounds (fluorescence intensities of CyPet-PRL2, CNNM3-YPet and a blank well). All data analysis was performed using GraphPad Prism 6 (GraphPad Software) with the methods described in each legend.

### Isothermal titration calorimetry

ITC experiments were performed by ITC200 (GE Healthcare, USA). PRL2 and CNNM3 CBS domain proteins were purified in Buffer A (20 mM Tris–HCl (pH 7.0), 50 mM NaCl, and 1 mM TCEP). JMS-053 (Aobious, USA) was dissolved in 100% DMSO at 10 mM, further diluted to 200 μM with Buffer A. The ITC cell was thermally equilibrated at 25 °C and then filled with 250 μl of 20 μM PRL2, while the syringe was filled with 40 μl of 200 μM JMS-053 or CNNM3 CBS protein. In the case of JMS-053, 2% DMSO at a final concentration was added into PRL2 protein solution. Data were analysed by Microcal Origin software.

### Ethical approval and informed consent

No human or vertebrate samples were used.

## Supplementary information

Supplementary Information.

## Data Availability

All data generated and analysed during the current study are available from the corresponding author. The plasmid constructs for the YPet-tagged CNNM3 CBS domain and CyPet-tagged PRL2 have been deposited into AddGene (https://www.addgene.org/) (Addgene IDs: 149686 and 149685).
