# Supplementary material for: A FRET-based screening method to detect potential inhibitors of the binding of CNNM3 to PRL2
Source: Sci Rep. 2020 Jul 30;10:12879. doi: 10.1038/s41598-020-69818-x (PMC7393355; doi:10.1038/s41598-020-69818-x)
Supplement: Supplementary file 1 — Supplementary Information. [file 41598_2020_69818_MOESM1_ESM.pdf]

A FRET-based screening method to detect potential inhibitors of the binding of CNNM3 to PRL2.

Faji Cai[1,2], Yichen Huang[1,2], Mengqi Wang[1], Minxuan Sun[1], Yimeng Zhao[1*], Motoyuki Hattori[1*]

[1]State Key Laboratory of Genetic Engineering, Collaborative Innovation Center of Genetics and Development, Department of Physiology and Biophysics, School of Life Sciences, Fudan University, 2005 Songhu Road, Yangpu District, Shanghai 200438, China. [2]These authors contributed equally to this work.

*Correspondence and requests for materials should be addressed to Y.Z. (email: ymzhao@fudan.edu.cn) or M.H. (email: hattorim@fudan.edu.cn).

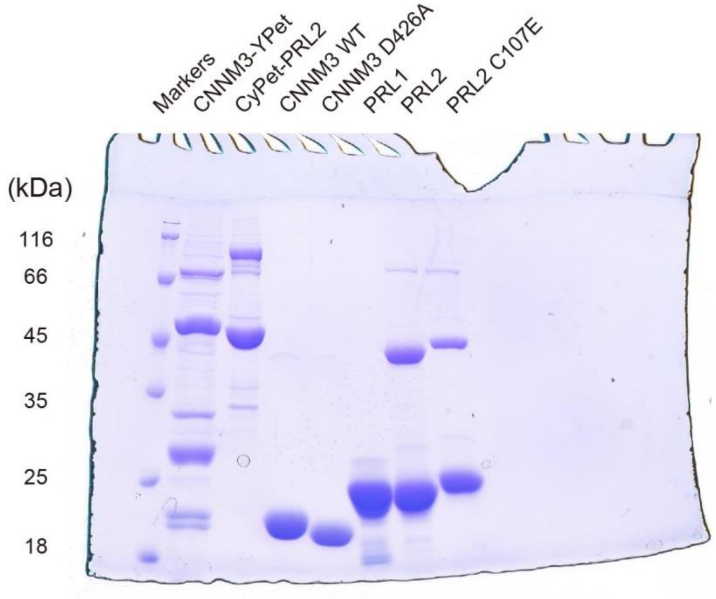

Supplementary Figure 1. SDS-PAGE analysis of purified proteins.

The first sample lane shows the markers, with the numbers indicating the molecular weights. The 2nd to 8th lanes are the purified CNNM3-YPet (MW: 47 kDa, purity: 31.6%), CyPet-PRL WT (MW: 49 kDa, purity: 78.8%), CNNM3 WT (MW: 20 kDa, purity: 96.3%), CNNM3 D426A (MW: 20 kDa, purity: 95.8%), PRL1 (MW: 23 kDa, purity: 84.8%), PRL2 WT (MW: 22 kDa, purity: 96.3%) and PRL2 C107E (MW: 22 kDa, purity: 93.3%). The PRL2 proteins seemingly formed partial dimers in the gel. Protein purities were estimated using ImageJ software. The gel image was not cropped.

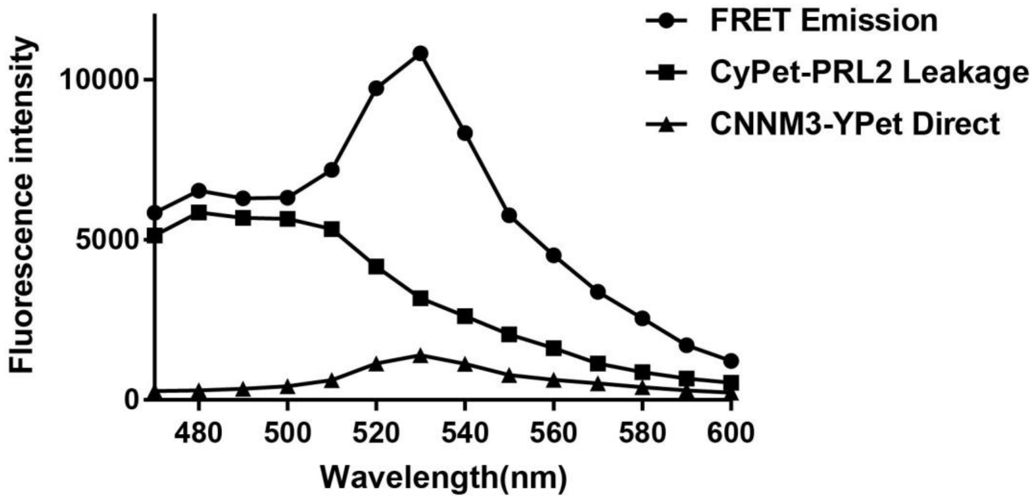

Supplementary Figure 2. Emission spectra of CyPet-PRL2/CNNM3-YPet.

Emission spectra of CyPet-PRL2/CNNM3-YPet (FRET Emission), CyPet-PRL2 and CNNM3-YPet excited at 435 nm. Averaged values from three independent measurements are presented. The FRET ratio [(FRET Emission – CyPet-PRL2 leakage)/CNNM3-YPet Direct] was approximately 5.5.

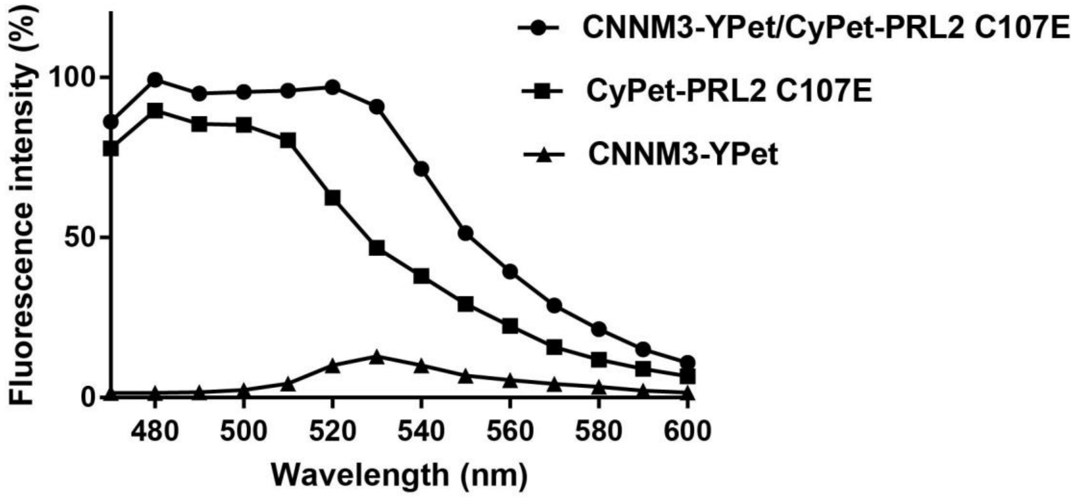

Supplementary Figure 3. Emission spectra of CyPet-PRL2 C107E/CNNM3-YPet.

Emission spectra of CyPet-PRL2 C107E/CNNM3-YPet, CyPet-PRL2 C107E and CNNM3-YPet excited at 435 nm. Averaged values from six independent measurements are presented. Each fluorescence intensity was normalized to the maximum values in the measurements.

Wavelength (nm)	FRET Emission			CyPet-PRL2 Leakage			CNNM3-YPet Direct		
470	4960	6210	6357	5161	5043	5198	269	277	279
480	5542	6915	7144	5974	5801	5775	293	301	294
490	5375	6761	6744	5775	5582	5707	345	344	340
500	5409	6710	6830	5751	5613	5607	394	419	447
510	6180	7569	7791	5396	5240	5368	565	625	640
520	8507	10291	10421	4210	4097	4198	994	1233	1169
530	9679	11314	11481	3251	3076	3207	1210	1515	1462
540	7435	8618	8946	2641	2593	2603	992	1213	1178
550	5049	6045	6198	2124	1997	2026	697	814	819
560	3912	4814	4827	1693	1641	1506	538	663	672
570	3102	3494	3530	1150	1120	1147	448	560	529
580	2262	2633	2734	857	879	873	368	417	412
590	1521	1800	1801	666	637	697	274	317	292
600	1116	1253	1289	517	551	533	214	238	208

Supplementary Table 1. Original data of the emission spectra for Supplementary Fig. 2.
